# Supplementary material for: Dynamic transcriptomic profiles of zebrafish gills in response to zinc supplementation
Source: BMC Genomics. 2010 Oct 11;11:553. doi: 10.1186/1471-2164-11-553 (PMC3091702; doi:10.1186/1471-2164-11-553)
Supplement: Additional file 2 — Interactive Direct Interaction Network representing the molecular interactions between zinc, copper, iron, calcium and proteins encoded by transcripts changed by zinc supplementation. Mini web-site containing index.html and hyperlinked pages in subdirectory describing a Direct Interaction Network automatically generated based on curated interactions contained within the proprietary PathwayArchitect database. Ovals represent proteins and the circles symbolize metal ions. Objects are coloured by their abundance in zebrafish at the time-point they were significantly different from the control is a scale from -4 fold (dark green) to +4 fold (dark red). Where significant differences were found at more than one time-point, the colour overlay shows expression at the first instance. Dark blue squares denote 'binding', and light blue squares 'expression'; green squares stand for 'regulation', green diamonds for 'metabolism', and green circles for 'promoter binding'. Arrow heads indicate directionality of the interaction where annotated. All nodes and edges can be further interrogated by selecting the relative area of the image. [file 1471-2164-11-553-S2.zip › PathwayArchitect Zn xs DIN/682115.html]

# REGULATION:

|  |  |
| --- | --- |
| Type | REGULATION |
| Effect | Positive |


---

|  |  |
| --- | --- |
| Score | 0 |


---

|  |  |
| --- | --- |
| Reference Count | 8 |


---

|  |  |
| --- | --- |
| Mechanism | Unknown |


---

|  |  |
| --- | --- |
| Reference:0 || Sentence | "Total iron absorption was further increased by heme iron absorption of 0.23 mg Fe/1000 kcal." |
| PMID | 12671125 |
| Year | 2003 |
| Species | Human |
| Journal | Pediatrics |
| RefScore | 0 |
| Source | PArchNLP |
  |
|  |  |


---

|  |  |
| --- | --- |
 Reference:1 || Sentence | "Iron dextran (Fe/Dex) was then administered parenterally to the animals, to induce iron overload." |
| PMID | 9067562 |
| Year | 1997 |
| Species | Human |
|  | Rat |
| Journal | Carcinogenesis |
| RefScore | 1 |
| Source | PArchNLP |
  ||  |  |


---

|  |  |
| --- | --- |
 Reference:2 || Sentence | "Addition of 45 mg Fe as Hb or NaFeEDTA to 0.4 mg Fe from Fe2(SO4)3 in the meal enhanced iron absorption from the latter in the same proportions." |
| PMID | 96688 |
| Year | 1978 |
| Species | Human |
| Journal | Am J Clin Nutr |
| RefScore | 1 |
| Source | PArchNLP |
  ||  |  |


---

|  |  |
| --- | --- |
 Reference:3 || Sentence | "Addition of 45 mg Fe as Fe2(SO4)3 and Hb to 0.4 mg Fe as NaFeEDTA in the meal respectively depressed and enhanced iron absorption from NaFeEDTA." |
| PMID | 96688 |
| Year | 1978 |
| Species | Human |
| Journal | Am J Clin Nutr |
| RefScore | 1 |
| Source | PArchNLP |
  ||  |  |


---

|  |  |
| --- | --- |
 Reference:4 || Sentence | "RESULTS: The mean S-Fe increase calculated as the area under the curve (AUC) from the reduced iron (RBV=0.43) and the electrolytic iron (RBV=0.73) differed significantly from the AUC following FeSO4 (p=0.002 and p=0.021, respectively)." |
| PMID | 14743958 |
| Year | 2003 |
| Species | Human |
| Journal | Scand J Clin Lab Invest |
| RefScore | 2 |
| Source | PArchNLP |
  ||  |  |


---

|  |  |
| --- | --- |
 Reference:5 || Sentence | "Cellular iron uptake is mediated by binding of transferrin with specific surface receptors and internalization of the Fe-transferrin-receptor complex." |
| PMID | 3162685 |
| Year | 1988 |
| Species | Human |
| Journal | Br J Haematol |
| RefScore | 0 |
| Source | PArchNLP |
  ||  |  |


---

|  |  |
| --- | --- |
 Reference:6 || Sentence | "The following four types of charge transfer are discussed in the light of our present resonance Raman data: (1) porphyrin (pi) leads to high-spin Fe (d pi), (2) azide (n) leads to low-spin iron (dz2), (3) azide (pi) leads to low-spin iron (dz2), and (4) azide (pi) leads to porphyrin (pi) (high spin)." |
| PMID | 7213625 |
| Year | 1981 |
| Species | Human |
| Journal | Biochemistry |
| RefScore | 3 |
| Source | PArchNLP |
  ||  |  |


---

|  |  |
| --- | --- |
 Reference:7 || Sentence | "Assembly of the Fe-S clusters is mediated by the iron-sulphate cluster assembly (ISC) machinery consisting of some ten proteins." |
| PMID | 10916152 |
| Year | 2000 |
| Species | Human |
| Journal | Trends Biochem Sci |
| RefScore | 0 |
| Source | PArchNLP |
  |


---

|  |  |
| --- | --- |
